# MicroRNA-3613-3p functions as a tumor suppressor and represents a novel therapeutic target in breast cancer

**DOI:** 10.1186/s13058-021-01389-9

**Published:** 2021-01-25

**Authors:** Chong Chen, Yundi Pan, Lipeng Bai, Huilin Chen, Zhaojun Duan, Qin Si, Ruizhe Zhu, Tsung-Hsien Chuang, Yunping Luo

**Affiliations:** 1 Department of Immunology, Institute of Basic Medical Sciences, Chinese Academy of Medical Sciences; School of Basic Medicine, Peking Union Medical College, Beijing, 100005 China; 2 Collaborative Innovation Center for Biotherapy, Institute of Basic Medical Sciences, Chinese Academy of Medical Sciences; School of Basic Medicine, Peking Union Medical College, Beijing, 100005 China; 3 Department of Clinical Laboratory, Jiangxi Cancer Hospital, Nanchang, 330029 China; 4 Immunology Research Center, National Health Research Institutes, Zhunan, Miaoli, Taiwan

**Keywords:** miR-3613-3p, Tumor suppressor, Tumor biomarker, Cancer cell proliferation, Cancer stem cell, Cell cycle

## Abstract

**Background:**

MicroRNAs have been reported to participate in tumorigenesis, treatment resistance, and tumor metastasis. Novel microRNAs need to be identified and investigated to guide the clinical prognosis or therapy for breast cancer.

**Method:**

The copy number variations (CNVs) of MIR3613 from Cancer Genome Atlas (TCGA) or Cancer Cell Line Encyclopedia (CCLE) were analyzed, and its correlation with breast cancer subtypes or prognosis was investigated. The expression level of miR-3613-3p in tumor tissues or serum of breast cancer patients was detected using in situ hybridization and qPCR. Gain-of-function studies were performed to determine the regulatory role of miR-3613-3p on proliferation, apoptosis, and tumor sphere formation of human breast cancer cells MDA-MB-231 or MCF-7. The effects of miR-3613-3p on tumor growth or metastasis in an immunocompromised mouse model of MDA-MB-231-luciferase were explored by intratumor injection of miR-3613-3p analogue. The target genes, interactive lncRNAs, and related signaling pathways of miR-3613-3p were identified by bioinformatic prediction and 3′-UTR assays.

**Results:**

We found that MIR3613 was frequently deleted in breast cancer genome and its deletion was correlated with the molecular typing, and an unfavorable prognosis in estrogen receptor-positive patients. MiR-3613-3p level was also dramatically lower in tumor tissues or serum of breast cancer patients. Gain-of-function studies revealed that miR-3613-3p could suppress proliferation and sphere formation and promote apoptosis in vitro and impeded tumor growth and metastasis in vivo. Additionally, miR-3613-3p might regulate cell cycle by targeting SMS, PAFAH1B2, or PDK3 to restrain tumor progression.

**Conclusion:**

Our findings indicate a suppressive role of miR-3613-3p in breast cancer progression, which may provide an innovative marker or treatment for breast cancer patients.

**Supplementary Information:**

The online version contains supplementary material available at 10.1186/s13058-021-01389-9.

## Introduction

Breast cancer is the most common cancer and the second cause of cancer-related death in women [1]. Improved treatments with combination of surgery, radiotherapy, and chemotherapy have increased the overall survival rate for breast cancer patients, but some patients still undergo tumor relapse, metastasis, or therapy resistance. Dysregulated proliferation is a typical feature of cancer cells and an essential target of cancer therapy [2]. Cancer stem cells (CSCs) possess a potent capacity for self-renewal and differentiation and, therefore, are responsible for the unrestricted growth of tumors [3]. Treatments specifically targeting CSCs may be a more effective strategy to eliminate the source of tumor growth, ultimately leading to considerable clinical benefit.

MicroRNAs (miRNAs) are endogenous, small non-coding RNAs that repress target genes expression by pairing to the 3′-untranslated regions (3′-UTR) of mRNAs [4]. A certain miRNA often has many targets and can participate in several pathways. miRNAs play a crucial role in various cancer and have potential applications in cancer diagnosis, prognosis, and therapy [5]. The strategy of miRNA-based therapies is either to restore tumor suppressive miRNAs or to block oncogenic miRNAs. Tumor suppressive miRNAs can be replenished by chemically modified miRNA mimics and oncogenic miRNAs can be reduced by complementary oligonucleotides, delivered by some novel systems like nanoparticles [6]. Some microRNA-based therapies have progressed into clinical trials [7]. Specially, different types or subtypes of cancer seem to have different miRNA expression profiles [8]. It is important to identify specific miRNAs associated with the tumor subtype when developing miRNA-targeted therapeutics.

Copy number variations (CNVs) are genomic amplifications or deletions, which are quite common in human genome and only a few of germline (or inherited) CNVs are associated with diseases [9]. Cancer harbors many de novo CNVs, called somatic CNVs. It has been demonstrated that amplification of oncogenic genes and deletion of tumor suppressor genes are relevant with tumorigenesis [10, 11]. Some somatic CNVs are common in cancer tissues and those frequently deleted are likely to contain tumor suppressor genes [12].

Hsa-mir-3613-3p was first identified through ultra-high throughput sequencing at moderate abundance in human cervical cells in 2010 [13]. A few studies have reported the change of mir-3613-3p associated with several types of cancer other than breast cancer, but the results are inconsistent and lack functional evidence [14–18]. In this study, we found that MIR3613 locus was frequently deleted in breast cancer tissues depending on whole exome sequencing data from The Cancer Genome Atlas (TCGA). It is important to note that MIR3613 deletion was associated with the breast cancer subtypes and the survival of estrogen receptor-positive (ER^+^) breast cancer patients. Moreover, miR-3613-3p level was also dramatically decreased in tumor tissues or serum of breast cancer patients. We also proved the tumor suppressive roles of mir-3613-3p in human breast cancer cell lines in vitro and in vivo. Furthermore, miR-3613-3p targets (coding or non-coding RNAs) were predicted by bioinformatic analysis and verified by biological experiments. These results elucidated the novel anti-tumor effect of mir-3613-3p in breast cancer.

## Methods

### Cell lines and human serum samples

The human breast cancer cell lines MDA-MB-231 and MCF-7, and human embryonic kidney cell line HEK293T, were obtained from American Type Culture Collection (ATCC) (October 2011) and cultured according to guidelines. The serum of breast cancer patients and healthy controls was obtained from Jiangxi Cancer Hospital and stored at − 20 °C.

### TCGA and CCLE database analysis

Gene-level copy number of miR-3613 was estimated by using the GISTIC2 method, and clinicopathological data for TCGA breast cancer cohort were retrieved from UCSC Xena (data downloaded on August 17, 2018) [19]. Gene-level copy number data for cancer cell lines of Broad Institute Cancer Cell Line Encyclopedia (CCLE) were retrieved from UCSC Xena (data downloaded on July 30, 2018) and were classified with a low-level threshold 0.3 and a high-level threshold 1 as described before [20, 21]. The significance of copy number variation was analyzed by GISTIC2.0, available through the Broad Institute TCGA copy number portal, filtered for *Q*-values less than 0.25 [12].

### In situ hybridization

The tissue microarray used for analysis of miR-3613-3p expression in breast cancer was purchased from Shanghai Outdo Biotech. miRCURY™ LNA™ Detection probe, 5′-DIG and 3′-DIG labeled (Art.No. YD00611450-BCG, Exiqon, Denmark), was used for in situ hybridization at a final concentration of 500 nM. The pictures of immunohistochemistry were captured by microscope (Leica, Germany).

### RNA isolation and quantitative real-time PCR

Total RNAs were harvested from cultured cells or animal tumor tissues using TRIzol (Invitrogen, USA) and a RNeasy Mini Kit (Qiagen, Germany) according to the manufacturer’s instructions. Serum miRNAs were harvested from 200 μL serum of each sample by using miRNeasy Serum/Plasma Kit (Catalog no. 217184, Qiagen, Germany) and miRNeasy Serum/Plasma Spike-In Control (Catalog no. 219610, Qiagen, Germany) was added as a normalization control.

Total RNA underwent reverse transcription using First Strand cDNA Synthesis Kit (Thermo Scientific technologies, USA) and each individual microRNAs’ sequence is shown in Supplementary Table 1. For microRNAs detection, stem–loop RT-PCR [22] was carried out by using an SYBR Green PCR master mix (TransGen Biotechnology, Beijing) and primer sequences are shown in Supplementary Table 2. All samples were normalized to internal controls and fold changes were calculated. Reverse transcription and real-time PCR of mir-3613-3p were also performed by using TaqMan probe (miR-3613-3p probe Art.No. 4427975, TaqMan), TaqMan MicroRNA Reverse Transcription Kit (Art.No. 4366596, TaqMan), and TaqMan Universal PCR Master Mix II (Art.No. 4440043, TaqMan) normalized to U6 small nuclear RNA (U6 probe Art.No. 4427975, TaqMan).

### MicroRNA transfection

miRNA mimic (Ribobio, China) were transfected using Lipofectamine 2000 (Invitrogen, USA) with a final miRNA concentration of 50 nM. Forty-eight hours after transfection, total RNA was isolated as before, and transfection efficiency was confirmed by miRNA qPCR.

### Cell proliferation

Forty-eight hours after transfection, MCF7 cells were plated in 96-well dishes at 6000 cells/well and MDA-MB-231 cells at 4000 cells/well. Cell growth rates were monitored using a Cell Counting Kit-8 (CCK-8) (Bimake, China) according to the manufacturer’s instructions.

### Tumor sphere assay

Single-cell suspensions were seeded in 6-well or 24-well ultra-low attachment plates (Corning, USA) in sphere-culturing medium (Stemcell Technologies, Canada) for 7 days. Tumor sphere formation was monitored using an inverted Leica microscope fitted with a camera as described previously [23] and CSCs frequency was analyzed by the extreme limiting dilution analysis (ELDA) online software [24].

### Apoptosis assay

Apoptosis was gauged 24 h post-transfection using FITC Annexin V Apoptosis Detection Kit II (BD, USA) according to the manufacturer’s instructions.

### Luciferase assay

To generate the luciferase reporter vectors, the 3′-untranslated regions (UTR) of genes of interest were synthesized and cloned into the pmirGLO Dual-Luciferase miRNA Target Expression Vector. Above distinct constructs with miRNA mimic were cotransfected into HEK293T or MCF-7 cells. Twenty-four hours after transfection, cells were analyzed for luciferase activity using the Dual-Glo® Luciferase Assay System (Promega, USA). Normalized firefly luciferase activity (firefly luciferase activity/Renilla luciferase activity, normalized to the pmirGLO Vector no-insert control) was compared. For each transfection, luciferase activity was averaged from five replicates.

### Bioinformatic analysis

For functional annotation, the database DAVID Bioinformatics Tool was used to search for the most enriched Kyoto Encyclopedia of Genes and Genomes (KEGG) pathways in selected gene lists. For target prediction, TargetScan, miRWalk, and miRTarBase were used to identify the mRNAs as miR-3613-3p targets [25–27]. DIANA-LncBase was used to identify the LncRNAs interacting with miR-3613-3p [28]. Gene Expression Profiling Interactive Analysis (GEPIA) was used to analyze the expression of candidate lncRNAs in breast cancer and normal tissues [29]. The prognostic value of each lncRNAs in breast cancer was analyzed by using Kaplan–Meier Plotter [30].

### Animal study

Female NOD/SCID mice, 6 to 8 weeks of age, were purchased from Vital River Laboratories. Approximately 1 × 10^6^ of luciferase-transfected MDA-MB-231 cells, mixed with Matrigel (1:1), were transplanted into 4th mammary gland fat pad of NOD/SCID mice. Mice were randomly divided into experimental groups with 5 mice in each group. After 12 days, 1 nmol of microRNA agomir (Ribobio, China) in 20 μl PBS was infused into the mammary gland once every 3 days for a total of 12 days (four times). Primary and metastatic tumors in mice were detected by PET scan (MicroPET Focus 120, Siemens, Germany). Lung tissues of each mice were fixed in formalin and embedded in paraffin for histologic analysis. There were four mice in each group in subsequent experiments.

### Statistical analysis

All quantified data represents an average of triplicate samples or as indicated. All experiments were repeated at least three times with similar results each time. Data are represented as mean ± S.E.M or as indicated. Comparisons between groups were analyzed with tow-tailed Student’s *t* test or Mann–Whitney test dependent on whether data conform to distribution normality. Chi-square test was used for correlation analyses between MIR3613 copy number and PAM50 subtypes and multiple comparison analysis was adjusted by Bonferroni correction. Pearson test was used for correlation analyses between primary tumor bioluminescence intensity and miR-3613-3p expression, which estimates a correlation value *r* and a significance *P* value (0 < *r* < 1, positive correlation; 0 > *r* > − 1, negative correlation). Overall survival of patients from the TCGA cohort was evaluated by the Kaplan–Meier method. Patients were divided into two groups based on the MIR3613 CNVs or the three target genes (SMS, PAFAH1B2, and PDK3) expression and the statistic differences of differentially expressed genes between the two groups were evaluated by the log-rank tests. Statistical analyses were performed using SPSS version 23. *P* < 0.05 was considered as the criterion for statistical significance (*P < 0.05; **P < 0.01; ***P < 0.001).

## Results

### MiR3613 locus was frequently deleted in breast cancer and associated with breast cancer subtypes and clinical prognosis

**Fig. 1:**
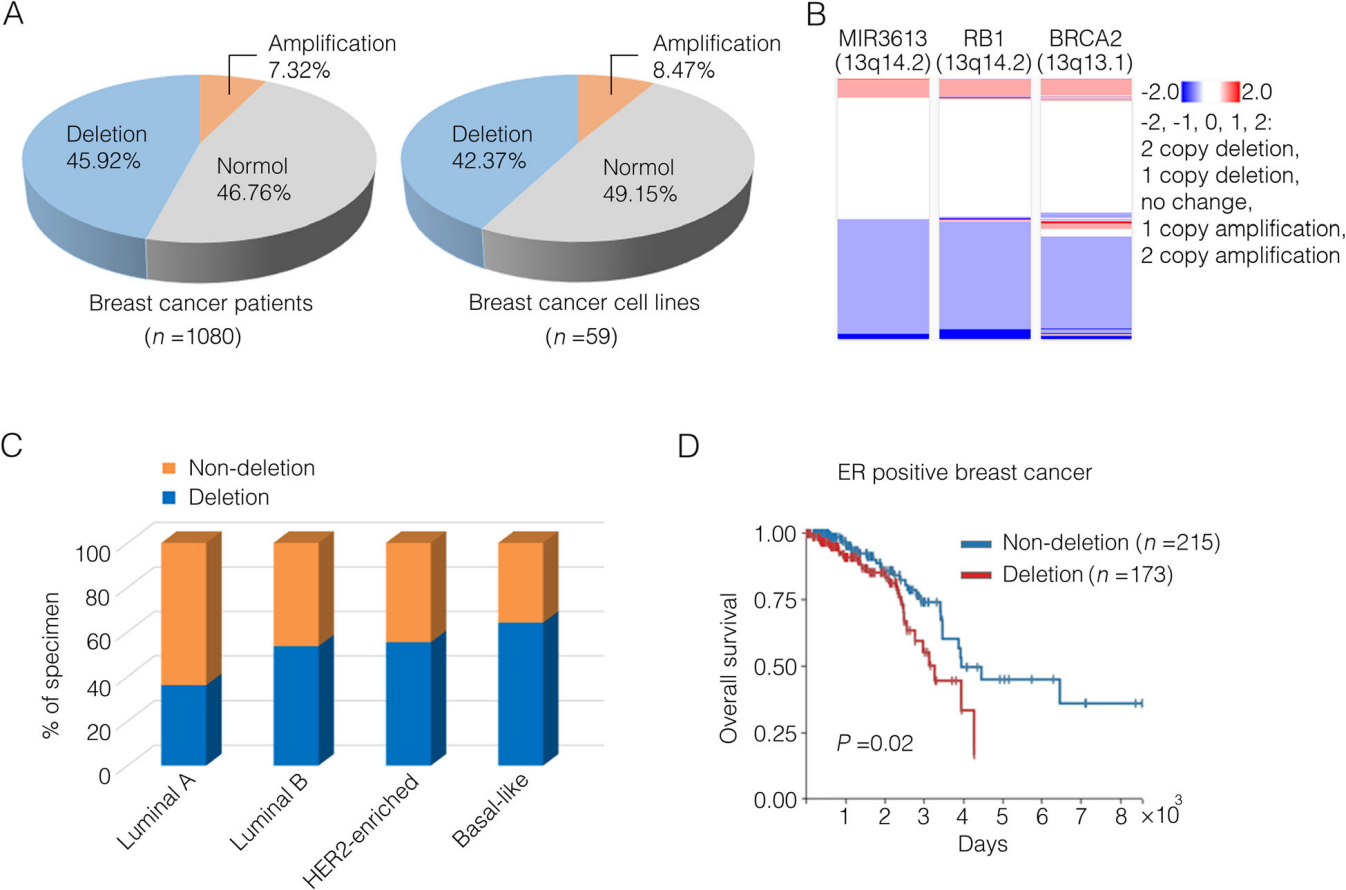
The relationship between copy number of miR-3613 and breast cancer subtypes and survival rate. **a** Left: Proportion description of diverse groups in breast cancer patients distinguished by their genomic copy number values (CNVs) of MIR3613 from TCGA database. Homozygous deletion or single copy deletion (CNV < 0) represented as deletion group, diploid normal copy (CNV = 0) represented as normal group, low-level copy amplification or high-level copy amplification (CNV > 0) represented as amplification group. Right: Proportion description of diverse groups in breast cancer cell lines distinguished by the MIR3613 CNV from Cancer Cell Line Encyclopedia (CCLE). **b** The CNVs of MIR3613, RB1 and BRCA2 in breast cancer patients from TCGA database. **c** MIR3613 CNV profiles in breast cancer subtypes from TCGA database. Different breast cancer subtypes contained diverse non-deletion (high-CNV) or deletion (low-CNV) patients’ distribution indicated by the Pearson chi-squared test (*P* < 0.001). Luminal A, *n* = 224; Luminal B, *n* = 127; HER2-enriched, *n* = 56; Basal-like, *n* = 92. **d** Kaplan–Meier survival curves of ER-positive breast cancer patients from TCGA database were depicted by MIR3613 CNVs (*P* = 0.02). The non-deletion group contained samples with high-CNV of MIR3613 (CNV ≥ 0, *n* = 215), while the deletion group contained samples with low-CNV of MIR3613 (CNV < 0, *n* = 173)

The copy number variations (CNVs) of MIR3613, which encoded miR-3613-3p, were analyzed across several cancer types in The Cancer Genome Atlas (TCGA) dataset using TCGA Copy Number Portal [12]. The locus of MIR3613 was significantly deleted across 13 diverse cancer types including breast invasive adenocarcinoma (Table 1). The proportion of MIR3613 deletions in TCGA breast cancer cohort was then studied using the UCSC Xena database [19]. Nearly 46% of TCGA breast cancer tumor samples were subjected to either heterozygous or homozygous deletions at MIR3613 locus and approximately 42% of breast cancer cell lines from Cancer Cell Line Encyclopedia (CCLE) showed copy number deletions of MIR3613 (Fig. 1a). Interestingly, MIR3613 locus (13q14.2) located near tumor suppressor genes RB1 (13q14.2) and BRCA2 (13q13.1) on chromosome 13 and copy number of this gene segment in breast cancer was frequently altered (Fig. 1b, Supplementary Fig. 1A) [12]. These results suggest that MIR3613 deletion is of common occurrence in breast cancer patients.

**Table 1:** miR-3613 is significantly deleted in 13 independent cancer types

Cancer type	*Q*-value	Overall frequency (%)
Prostate adenocarcinoma	2.41×10^-47^	44.51
Bladder urothelial carcinoma	1.78×10^-29^	37.01
Liver hepatocellular carcinoma	8.42×10^-23^	48.11
Glial cancers	1.34×10^-17^	36.06
Breast invasive adenocarcinoma	1.29×10^-16^	46.11
Glioblastoma multiforme	3.7×10^-12^	42.98
Cervical squamous cell carcinoma	3.5×10^-11^	38.98
Head and neck squamous cell carcinoma	2.55×10^-7^	40.42
Brain lower grade glioma	2.23×10^-5^	28.27
Ovarian serous cystadenocarcinoma	2.63×10^-5^	62.52
Lung squamous cell carcinoma	4.49×10^-3^	68.06
Lung adenocarcinoma	1.84×10^-2^	53.88
Diffuse large B cell lymphoma	1.3×10^-1^	14.58

Significance (*Q*-value): Low *Q*-values (< 0.25) suggest that deletions at this locus are enriched by selective pressures and this locus has a possible role in cancer initiation, growth, or survival. Overall frequency: Overall frequency measures the fraction of cancers which exhibit any deletion at this locus

The genomic study in 2012 gave a chance to examine the relationship between miR-3613 copy numbers and PAM50 subtypes [31]. The PAM50 gene signature classifies breast cancer into 4 intrinsic subtypes according to 50 genes expression [32]. A total of 499 cancer samples from TCGA dataset having PAM50 subtype identities were divided into the non-deletion (CNV ≥ 0, *n* = 254) group and the deletion (CNV < 0, *n* = 232) group according to whether there was a deletion of MIR3613. The luminal A subtype specifically contained more non-deletion samples and the basal-like subtype contained more deletion samples (Fig. 1c). Although no significant correlation was observed between MIR3613 copy number and survival in the whole cohort or ER negative subset of breast cancer patients (Supplementary Fig. 1B), deletion of MIR3613 was associated with an unfavorable prognosis in the ER positive patients (Fig. 1d). Taken together, these results suggest that MIR3613 genomic loss is related to poor prognosis in breast cancer patients.

### MiR-3613-3p is downregulated in tumor tissues or serum of breast cancer patients

**Fig. 2:**
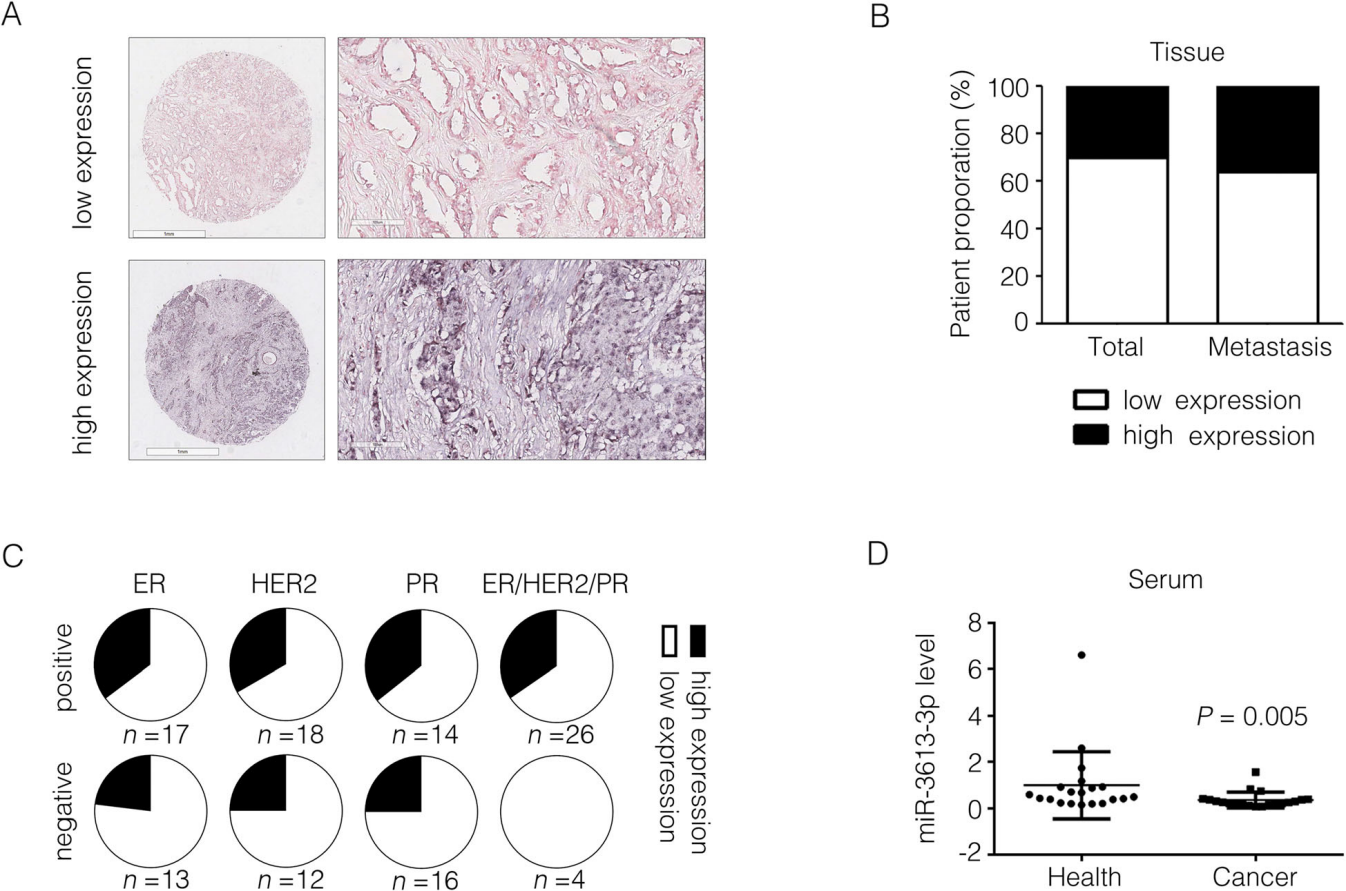
miR-3613-3p expression in clinical breast cancer patients. **a** miR-3613-3p was detected by in situ hybridization and representative images of low or high expression from breast cancer tissue array were shown. **b** The proportion of patients with lymph node metastasis or not was calculated depending on miR-3613-3p expression. **c** Fractions of two group patients divided based on the miR-3613-3p expression were analyzed in different subtypes of breast cancer from breast cancer tissue array. **d** miR-3613-3p in serum of breast cancer patients was detected by RT-PCR (*P* = 0.005)

To further evaluate its clinical relevance, miR-3613-3p expression in breast cancer tissues from 30 individual breast cancer patients was detected by using in situ hybridization and their clinical information was shown in Supplementary Table 3. These patients were divided into a miR-3613-3p low expression group and a miR-3613-3p high expression group and the representative images were shown in Fig. 2a (in situ hybridization) and Supplementary Fig. 2 (H&E). Low expression of miR-3613-3p was shown in 21 out of all 30 breast cancer patients (accounting for 70%) and in 9 out of 14 patients having lymph node metastasis (accounting for 64%) which demonstrated a conspicuously lower expression of miR-3613-3p in cancer tissues (Fig. 2b). In accord with the results from TCGA database (Fig. 1c), patients of miR-3613-3p low-expression from breast cancer tissue array accounted for a larger proportion in patients with negative clinical marker (ER, HER2 or PR) accordingly compared to patients with positive clinical marker (Fig. 2c). Besides that, we also collected blood serum samples from other 20 breast cancer patients, and 20 healthy women and their clinical information are shown in Supplementary Table 4. It was noteworthy that the expression of miR-3613-3p in serum were dramatically decreased in cancer samples detected by RT-PCR (Fig. 2d). Taken together, these findings suggest that miR-3613-3p is distinctly downregulated in clinical samples of breast cancer patients.

### MiR-3613-3p suppressed malignant phenotypes of breast cancer cells in vitro

**Fig. 3:**
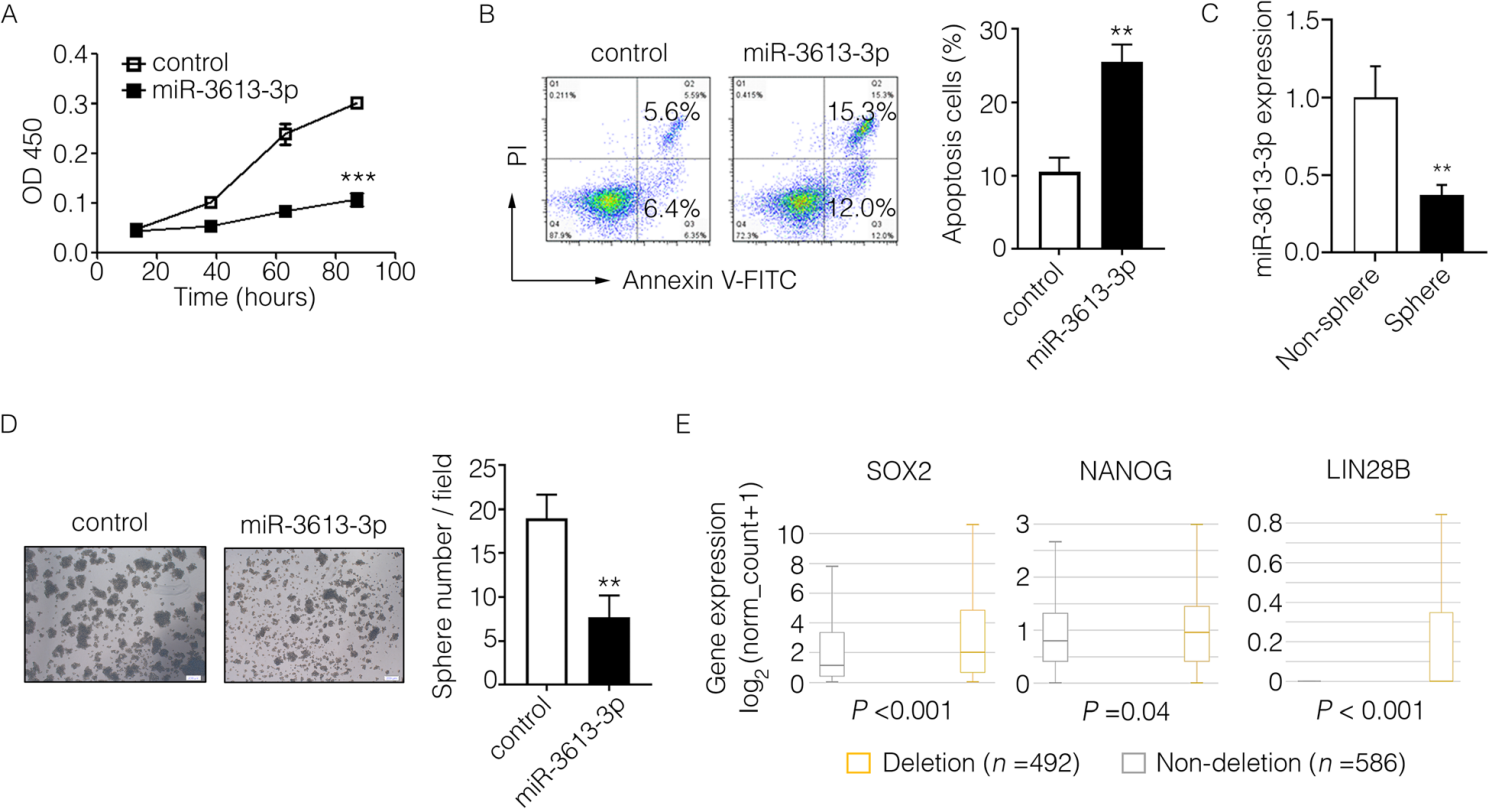
miR-3613-3p suppressed malignant phenotype of human breast cancer cells. **a** Proliferation of MCF7 cells transfected by miR-3613-3p mimic or control mimic was analyzed by CCK-8 assay (***, *P* < 0.001). **b** Apoptosis of MDA-MB-231 cells transfected by miR-3613-3p mimic or control mimic was analyzed by flow cytometry. The representative apoptosis images were visualized on the left and the proportion of apoptosis cells were statistically analyzed on the right (**P < 0.05). **c** The expression of miR-3613-3p in non-spheres or spheres of MDA-MB-231 cells was calculated by RT-PCR (**P < 0.05). **d** Tumor spheres of MDA-MB-231 cells transfected by miR-3613-3p mimic or control mimic was cultured with serum-free medium for 7 days. The representative tumor sphere images were visualized by microscope on the left (scale bar, 200 mm) and the sphere number per field were statistically analyzed on the right (**P < 0.05). **e** Transcripts of differentially expressed genes were analyzed in two groups of breast cancer patients divided according to MIR3613 CNVs from TCGA dataset (group of MIR3613 CNV ≥ 0, MIR3613 non-deletion, *n* = 586; group of MIR3613 CNV< 0, MIR3613 deletion, *n* = 492). The expression of SOX2, NANOG, and LIN28B in above two groups were shown according to the transcriptome sequencing data

To address whether miR-3613-3p might play a key role in malignant characters in breast cancer cells, we first measured the proliferation or apoptosis or human breast cancer cell lines MCF7 and MDA-MB-231 cells transfected with miR-3613-3p or control mimics, respectively. After overexpression of miR-3613-3p, the proliferation rate of MCF7 cells was significantly suppressed (Fig. 3a), whereas the proportion of apoptotic cells was markedly increased in MDA-MB-231 cells (Fig. 3b). However, neither proliferation rate of MDA-MB-231 cells nor the apoptotic proportion of MCF7 cells was changed after miR-3613-3p overexpression (Supplementary Fig. 3A, B). Tumor spheres forming ability was a golden standard to evaluate the self-renewal capacity of cancer stem cells (CSCs). After culturing and separating of tumor spheres, we found that stemness associated genes (SOX2, OCT4, NANOG, and LIN28B) exhibited a high level of expression whereas stemness-suppressive microRNAs (let7 family and miR-146a) exhibited a low level of expression in spheres in accordance with previous studies (Supplementary Fig. 4A, B). Interestingly, we observed that miR-3613-3p expression was dramatically decreased in tumor spheres (Fig. 3c) and it could obviously suppress the tumor spheres forming ability of MDA-MB-231 cells as well (Fig. 3d, Supplementary Fig. 5A, B), indicating the inhibitory effect of miR-3613-3p in cancer stemness maintenance. We then analyzed the transcripts of differentially expressed genes in two groups of breast cancer patients divided according to MIR3613 CNVs from TCGA dataset (group of MIR3613 CNV ≥ 0, MIR3613 non-deletion, *n* = 586; group of MIR3613 CNV < 0, MIR3613 deletion, *n* = 492). Interestingly, the expression of stemness genes (SOX2, NANOG, and LIN28B) were all significantly higher in the MIR3613 deletion group compared to the non-deletion group (Fig. 3e). These results suggest that miR-3613-3p can suppress the malignant phenotypes and cancer stemness of breast cancer cells and may serve as a tumor suppressor.

### MiR-3613-3p suppressed tumor growth and metastasis in an immunocompromised mouse model of human breast cancer cell MDA-MB-231

**Fig. 4:**
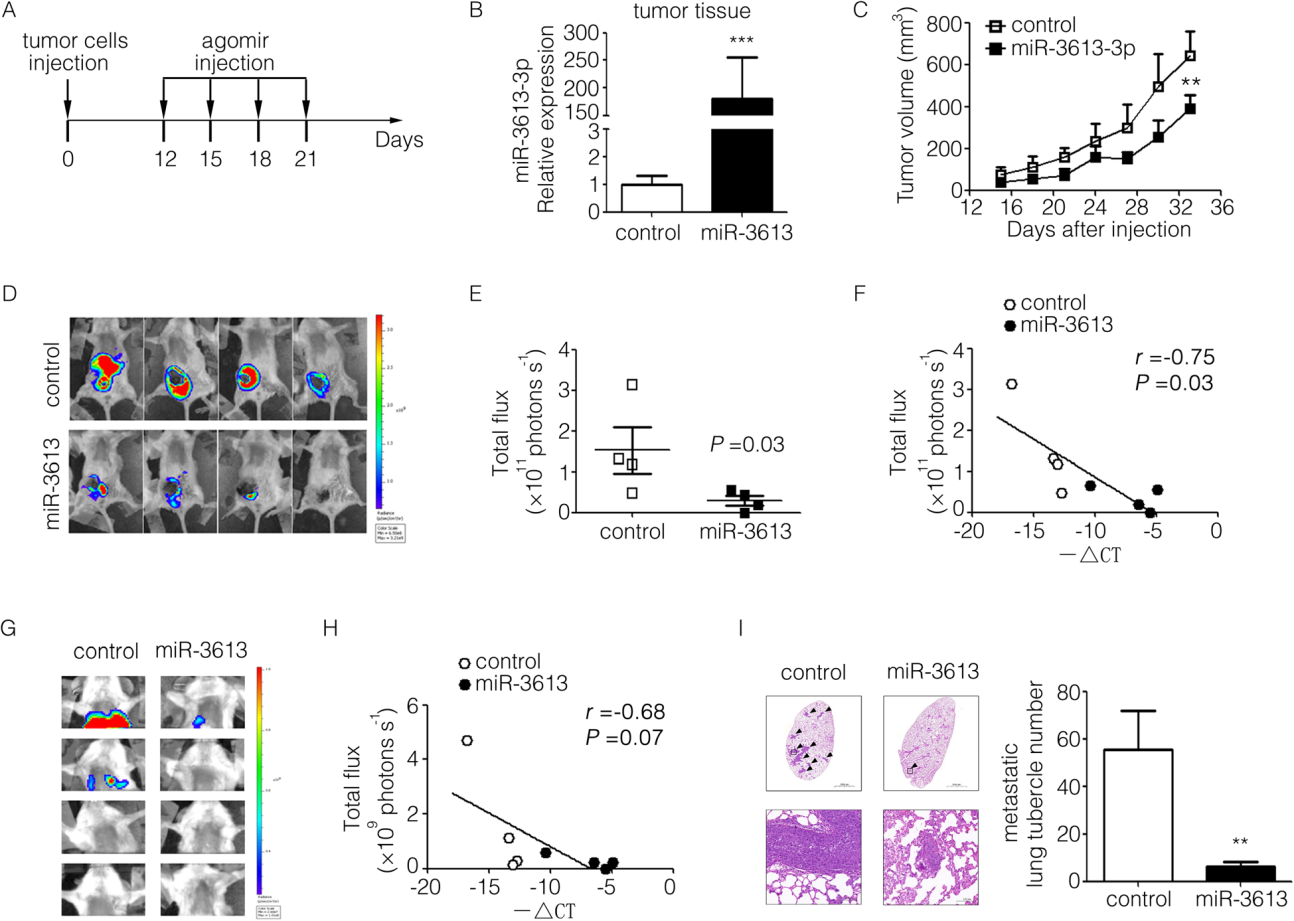
miR-3613-3p suppressed tumor growth and metastasis of MDA-MB-231 cells in NOD/SCID mice. **a** The strategy of animal experiment was illustrated. After tumor volume reached about 50 mm^3^, miR-3613-3p or control agomir was intratumor injected respectively every 2 days for four times. **b** The expression of miR-3613-3p in tumor tissues from miR-3613-3p or control group were detected by RT-PCR after mice were killed on day 60 (***P < 0.001). **c** Tumor growth curve of mice injected with miR-3613-3p or control agomir (Shown are mean tumor volumes ± SEM; *n* = 4 per group; **P < 0.05). **d** Bioluminescence images of the primary tumors from miR-3613-3p or control mice were captured by Live animal imager on day 59. **e** Bioluminescence intensity of primary tumors were statistically analyzed (*P* = 0.03). **f** Correlation between primary tumor bioluminescence intensity and miR-3613-3p expression was calculated by Pearson test (*r* = − 0.75, *P* = 0.03). **g** Bioluminescence images of the pulmonary metastases were captured by Live animal imager on day 59. **h** Correlation between primary tumor bioluminescence intensity and miR-3613-3p expression was calculated by Pearson test (*r* = − 0.68, *P* = 0.07). **i** Representative images of the pulmonary metastases in mice by H&E staining (arrow marked typical metastases; Scale bar, 100 mm). **j** Metastatic lung tubercle numbers were calculated from H&E staining images (**P < 0.05)

To investigate the effect of miR-3613-3p on tumor progression in vivo, luciferase-labeled MDA-MB-231 cells were injected into the 4th mammary gland fat pad of NOD/SCID mice. Agomir, a chemically modified analogue of microRNA with enhanced stability and activity, was successfully used to explore the function of microRNA in vivo previously [33]. On the 12th day after tumor inoculation, miR-3613-3p agomir or negative control agomir were intratumorally injected respectively and tumor growth or lung metastasis was monitored (Fig. 4a). Results showed that the expression of miR-3613-3p in tumor was increased as expected (Fig. 4b) and volumes of tumor in situ were dramatically reduced by administrating of miR-3613-3p agomir (Fig. 4c). In fact, primary tumor growth in groups of mice given by miR-3613-3p agomir was clearly delayed as indicated by living imaging and the signal intensity in primary tumor cells of mice was negatively correlated with miR-3613-3p expression (*r* = − 0.75, *P* = 0.03) (Fig. 4d–f). Furthermore, the degree of pulmonary metastasis in the miR-3613-3p group significantly reduced and the signal intensity in pulmonary tumor cells of mice was slightly negatively correlated with miR-3613-3p expression (*r* = − 0.68, *P* = 0.07) (Fig. 4g, h). H&E staining of lung specimens indicated obviously that the number of metastatic foci number in lung were much fewer in groups of mice subjected to miR-3613-3p agomir treatment (Fig. 4i). Taken together, these results reveal a remarkable role of miR-3613-3p in tumor suppression in vivo and likely provide a useful target for future therapeutic interventions in breast cancer.

### Identification of miR-3613-3p target genes and related signaling pathway

**Fig. 5:**
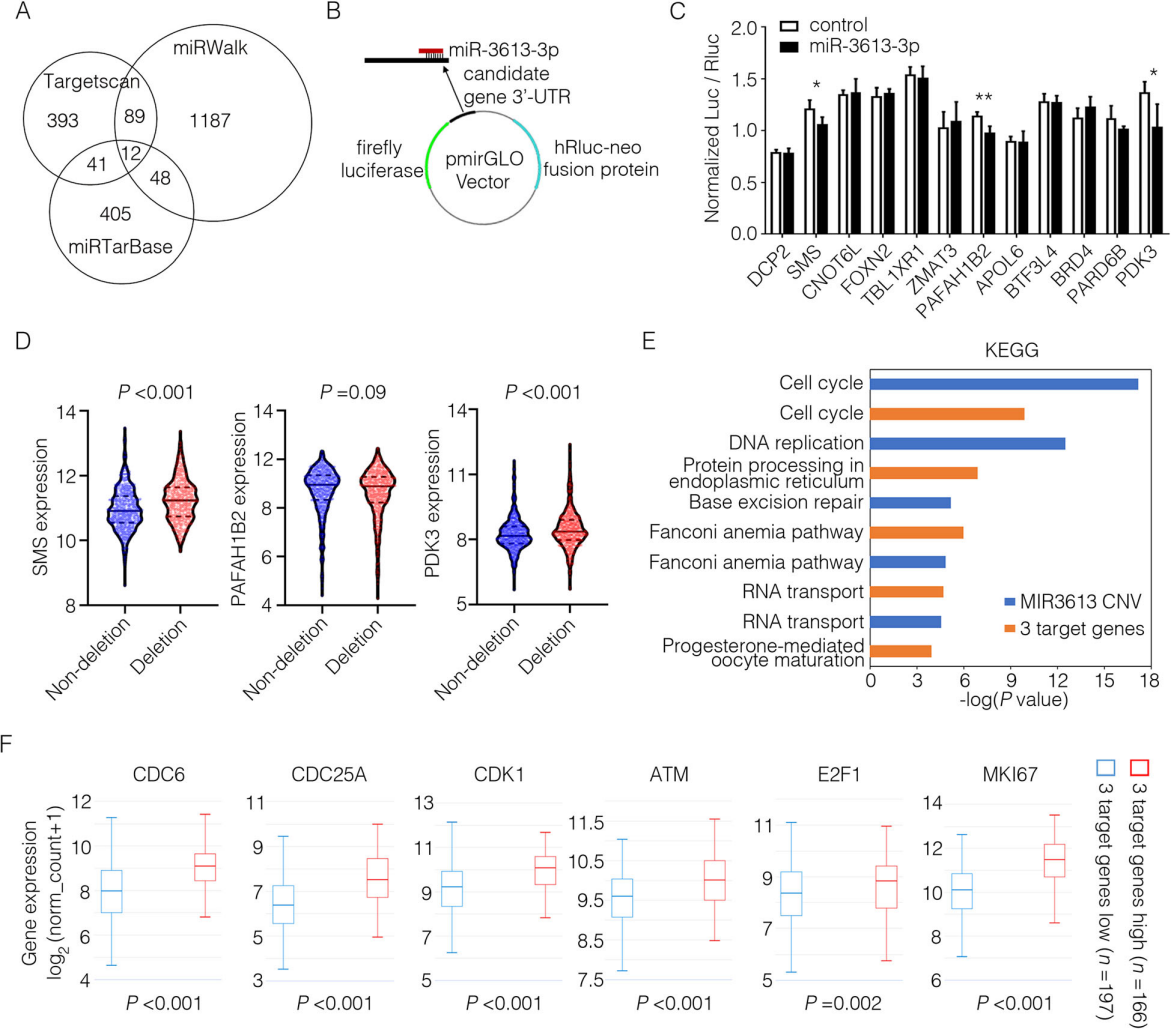
Identification of miR-3613-3p target genes and related signaling pathway. **a** Venn diagram of predicted target genes of miR-3613-3p from Targetscan, miRWalk and miRTarBase online websites. **b** Schematic diagram of 3′-UTR dual-luciferase reporter system. **c** Predicted 12 target genes of miR-3613-3p were verified by 3′-UTR dual-luciferase reporter assays in HEK293T or MCF-7 cells (*P < 0.05). **d** The expression of SMS, PAFAH1B2, or SNHG16 was shown in two separate group samples (MIR3613 deletion group or MIR3613 non-deletion group) of breast cancer tissues from TCGA database. **e** Transcripts of differentially expressed genes were analyzed in two groups of breast cancer patients divided according to MIR3613 CNVs from TCGA dataset (group of MIR3613 CNV ≥ 0, *n* = 586; group of MIR3613 CNV < 0, *n* = 492), and enriched top five signaling pathways (blue) were shown. Transcripts of differentially expressed genes were analyzed in two groups of breast cancer patients divided according to SMS, PAFAH1B2, and PDK3 expression from TCGA dataset (group of 3 target genes high expression, *n* = 166; group of 3 target genes low expression, *n* = 197), and enriched top five signaling pathways (orange) were shown. **f** Transcripts of differentially expressed genes were analyzed in two groups of breast cancer patients divided according to SMS, PAFAH1B2, and PDK3 expression from TCGA dataset (group of 3 target genes high expression, *n* = 166; group of 3 target genes low expression, *n* = 197). The expression of CDC6, CDC25A, CDK1, ATM, E2F1, and MKI67 in above two groups were shown according to the transcriptome sequencing data

To identify the mechanism(s) whereby miR-3613-3p exerted its biological effect on breast cancer cells, we first ran a target prediction in silico using TargetScan, miRWalk and miRTarBase. From 7395 target genes predicted by TargetScan, we selected 535 genes with the highest absolute value of context score which indicated as the probability of targeting. Functional pathway analysis revealed that the shared target genes predicted by TargetScan and miRWalk (*n* = 101) were mainly enriched in cancer-related signaling pathways (Supplementary Fig. 6A, B). Furthermore, 12 of miR-3613-3p target genes were identified as the overlaps among those predicted by TargetScan, miRWalk, and miRTarBase (Fig. 5a). Then, we constructed the dual-luciferase reporter system of 3′-untranslated region (UTR) from these 12 target genes to identify miR-3613-3p targets. To generate the luciferase reporter vectors, gene fragments, which are approximately 400 to 800 base pairs, including the predicted combining loci in the 3′-UTR, were synthesized and cloned into the pmirGLO Dual-Luciferase miRNA Target Expression Vector (Fig. 5b). Decreased luciferase activity was found in constructs for three genes (SMS, PAFAH1B2, and PDK3) in HEK293T or MCF-7 cells (Fig. 5c) and their binding sites with miR-3613-3p are shown in Supplementary Fig. 11A. More importantly, the highly amplified transcripts of SMS and PDK3 were observed in breast cancer samples with miR-3613 deletion compared to the non-deletion group from TCGA breast cancer cohort (Fig. 5d). We then analyzed the signaling pathways potentially regulated by these 3 target genes or miR-3613-3p. On the one hand, differentially expressed genes and enriched signaling pathways were analyzed in two groups of breast cancer patients divided according to MIR3613 CNVs from TCGA dataset (group of MIR3613 CNV ≥ 0, *n* = 586; group of MIR3613 CNV < 0, *n* = 492). On the other hand, differentially expressed genes and enriched signaling pathways were analyzed in two groups of breast cancer patients divided according to SMS, PAFAH1B2, and PDK3 expression from TCGA dataset (group of 3 target genes high expression, *n* = 166; group of 3 target genes low expression, *n* = 197). It is worth noting that among the top 10 of KEGG signaling pathways, cell cycle was the most relevant pathway in both two-independent analysis (Fig. 5e, Supplementary Fig. 7). Further analysis demonstrated that the expression of cell cycle-related genes (CDC6, CDC25A, CDK1, ATM, E2F1, and MKI67) were much higher in breast cancer patients of 3 target genes high expression group or MIR3613 deletion group compared with their counterpart, respectively (Fig. 5f, Supplementary Fig. 8).

The overall survival rates of breast cancer patients based on the expression of these three genes (SMS, PAFAH1B2 and PDK3) were analyzed by UCSC Xena (data retrieved on November 24, 2020) [19]. In all the patients, regardless of ER status, high expression of PAFAH1B2 (*P* = 0.04), but not SMS (*P* = 0.35) or PDK3 (*P* = 0.23), was associated with an unfavorable prognosis (Supplementary Fig. 9A-C). In ER-positive patients, high expression of PAFAH1B2 (*P* = 0.02) or PDK3 (*P* = 0.04) indicated a poor prognosis and patients with high expression of SMS (*P* = 0.09) showed a tendency towards a worse overall survival (Supplementary Fig. 9A-C). However, in ER-negative patients, no significant correlation was observed between the expression of these three genes and survival rates (Supplementary Fig. 9A-C). Furthermore, in the ER-positive subsets, but not in the whole subsets or ER-negative subsets, patients with simultaneously high expression of all these three genes had markedly poorer prognosis compared to patients with low expression of all of them (*P* = 0.03, Supplementary Fig. 9D). These results strongly suggested that higher level of SMS, PAFAH1B2, or PDK3 could predict worse prognosis in ER-positive breast cancer.

However, the specific biological functions of SMS, PAFAH1B2, or PDK3 need to be further investigated in breast cancer. These results suggest that miR-3613-3p perhaps regulate cell cycling pathway by targeting SMS, PAFAH1B2, or PDK3 to restrain tumor progression.

### Bioinformatic analysis of lncRNAs interacting with miR-3613-3p

Long non-coding RNAs (lncRNAs) have been reported to play important roles in carcinogenesis and acting as a sponge for several miRNAs in previous studies [34]. To investigate the specific lncRNAs interacting with miR-3613-3p, the online software DIANA-LncBase (https://omictools.com/diana-lncbase-tool) was used, which offered miRNA–lncRNA interactions supported by either computational prediction or experimental verification. Forty-one lncRNAs were both experimentally supported and in silico predicted targets, in which 6 were predicted targets specifically in breast tissue (Supplementary Fig. 10A). Then, we explored the expression levels of these 41 lncRNAs in breast cancer and their association with overall survival using the GEPIA database and the Kaplan–Meier Plotter database. Out of the 30 available expression results, 7 lncRNAs had lower expression levels in breast cancer tissues than in normal breast tissue (Supplementary Fig. 10B). Out of the 13 available survival curve results, low expression of NEAT1 or high expression of SNHG16 was associated with significantly shorter survival in 626 breast cancer patients (Supplementary Fig. 10C). The binding sites of NEAT1 and SNHG16 with miR-3613-3p are shown in Supplementary Fig. 11B, respectively. It is noteworthy that NEAT1 was decreased and SNHG16 was increased in breast cancer samples with MIR3613 deletion compared to the non-deletion group from TCGA breast cancer cohort (Supplementary Fig. 10D). These data potentially indicate that NEAT1 or SNHG16 may interact with miR-3613-3p and perform their diverse biological functions in breast cancer.

## Discussion

MicroRNAs, as an important class of non-coding small RNAs, almost affect all the cellular processes and have been found to be dysregulated in a multitude of diseases. The study of Calin et al. reveals that miRNA loci are frequently located in fragile sites associated with cancers [35]. The role of genes at fragile genomic locations is controversial, but emerging evidence support that a lot of these genes contribute to tumorigenesis [36, 37]. Non-coding genes may have critical functions, as well as protein-coding genes. Mir-101 is often deleted in non-small cell lung cancer and displays anti-tumorigenic properties [38]. Mir-383-3p is at a frequently missing genomic locus in prostate cancer and impedes cancer initiation and metastasis [39]. In the present study, we first found a widespread loss of miR-3613-3p DNA fragment (13q14.2) in breast cancer patients genome, which located near the famous tumor suppressor genes RB1 (13q14.2) and BRCA2 (13q13.1). It is coincident that miR-15/16 located to 13q14 were also deleted in patients with chronic lymphocytic leukemia [40]. PAM50 signature assay has displayed additive values in breast cancer prognosis and treatment decisions combined with standard clinical and pathological factors [41]. Interestingly, a lower frequency of MIR3613 deletion was most restricted in PAM50 subtypes with a comparatively favorable prognosis (luminal A subtype) and ER-positive breast cancer patients with MIR3613 deletion possessed a worse prognosis compared with the non-deletion group. Previously, our group identified serum miR-1915-3p and miR-455-3p as biomarkers for breast cancer patients with lymph node metastasis [42]. This study further revealed a lower expression of miR-3613-3p in the blood serum of breast cancer patients compared with healthy controls and a decreased level was also shown in breast cancer tissues of patients with or without metastasis. These results strongly imply that miR-3613-3p serves as a molecular indicator or tumor suppressor for breast cancer initiation and progression.

Uncontrolled proliferation is an essential characteristic of cancer and interruption to proliferative pathways is a promising strategy to fight cancer [3]. Overexpression of miR-3613-3p significantly impairs the proliferation and promotes the apoptosis of breast cancer cells in vitro. In line with our data, Zhang et al. recently discovered miR-3613-3p could inhibit hepatoma cell proliferation [43]. However, our data revealed that overexpression of miR-3613-3p had almost no influence on the proliferation of MDA-MB-231 cells and the apoptosis of MCF-7 cells. MCF7 (ER positive) and MDA-MB-231 (ER negative) cells had different molecular pathways impacting their distinct malignant behaviors [44]. Since miR-3613-3p could target multiple genes, it was probable that the major targets or relative molecular pathways of miR-3613-3p in ER-positive and ER-negative breast cancers were not the same. In addition, a BRCA2 mutation was frequently observed to associate with worse prognosis in ER-positive breast cancer and the basic mechanism was still unclear [45]. Our results suggested that MIR3613 locus (13q14.2) located near BRCA2 (13q13.1) in breast cancer and they perhaps had some functional similarity and interactivity.

In our previous studies, we demonstrated that targeting CSCs or CSC-associated microRNA (miR-34a) could markedly improve the efficacy of cancer therapy [23, 46]. In this study, the expression of miR-3613-3p was decreased in tumor spheres and it could dramatically inhibit the self-renewal of CSCs (tumor sphere forming ability), which suggested a novel suppressive function of miR-3613-3p in CSCs. It should be noticed that some microRNAs regulating cancer stemness were supposed as excellent therapeutic targets in previous studies [47, 48]. Importantly, miR-3613-3p repressed tumor growth and metastasis in an immunocompromised mouse model of human breast cancer cell MDA-MB-231. Molecular and histologic analysis of tumor tissues has provided microscopic evidence that the expression of miR-3613-3p was inversely correlated with tumor progression. Although cell migratory capacity did not change much in vitro, the pulmonary metastasis was dramatically suppressed by miR-3613-3p agomir in mice, implying the complexity of metastatic process in vivo [49]. These phenotype findings are consistent with previous genomic studies suggesting that loss-function of miR-3613-3p is an important mechanism in breast cancer progression and thus provide a novel therapeutic target.

Functional annotation revealed that predicted target genes of miR-3613-3p were mostly enriched in cancer related pathways. Bioinformatic prediction and 3′-UTR assay identified SMS, PAFAH1B2, and PDK3 could be regulated by miR-3613-3p. Spermine synthase, encoded by SMS, catalyzed polyamine metabolism, whose dysregulation was associated with carcinogenesis [50]. High SMS mRNA expression was related to poor survival and increased risk of metastasis in triple-negative breast cancer [51]. PAFAH1B2 encodes a subunit of platelet-activating factor acetyl hydrolase and was identified to participate in metabolic cancer pathogenicity [52]. Blockade of PAFAH1B2 and PAFAH1B3 causes a heightened level of tumor-suppressing lipids and damages cancer pathogenicity in breast cancer and several other types of cancer. Pyruvate dehydrogenase kinase 3 (PDK3) could alter glucose metabolism in cancer by inactivating pyruvate dehydrogenase kinase and overexpression of PDK is related to tumor invasion, metastasis, and drug resistance [53]. It is interesting to note that cell cycle, important for cell proliferation, was the most enriched signaling pathway regulated by these three target genes or miR-3613-3p, which mightily suggested miR-3613-3p targeted SMS, PAFAH1B2, or PDK3 to suppress oncogenic pathway of cell cycle for tumor control. Recently, miR-3613-3p was reported to regulate genes of EGFR signaling pathway in the epithelial-mesenchymal transition of lung adenocarcinoma [54]. It is possible that miR-3613-3p affect cancer pathogenicity in different ways in diverse tumor types and further investigation need to be complete investigation. Furthermore, seven lncRNAs, downregulated in breast cancer tissues and potentially interacting with miR-3613-3p, were identified by bioinformatic analysis. The expression of NEAT1 was markedly decreased in MIR3613 deletion group and its high expression tended to be associated with prolonged survival in breast cancer patients. Previous study revealed that NEAT1 emerged as an important regulator in cancer development and was downregulated in invasive breast cancer [55], which was consistent with our results. SNHG16, upregulated in miR-3613 deletion group, may act as a sponge for miR-3613-3p and its high expression was associated with a worse prognosis in breast cancer patients. The biological functions of NEAT1 and SNHG16 are rarely investigated in breast cancer and our findings provided a novel direction in the field of cancer-related lncRNAs for future research.

## Conclusions

In this study, we discovered the frequent genomic deletion and low expression of miR-3613-3p in breast cancer for the first time. In vitro and in vivo experimental validation proved the important suppressive role of miR-3613-3p in breast cancer progression. Bioinformatic analysis revealed miR-3613-3p might regulate cell cycle signaling pathway by targeting SMS, PAFAH1B2, or PDK3 to restrain tumor progression. Our findings imply the important role of miR-3613-3p as an innovative prognosis marker or treatment target for breast cancer patients.

## Supplementary Information

**Additional file 1: Supplementary Figure 1.** The genomic location of MIR3613 and its CNV in breast cancer patients prognosis. A) The genomic location of MIR3613, RB1 and BRCA2 (red lines): bands according to Ensembl, locations according to GeneLoc. B) Kaplan–Meier survival curves of breast cancer patients from TCGA database were depicted by their genomic copy number value (CNV) of miR-3613 (*P* = 0.18). The non-deletion group contained samples with high-CNV miR-3613 (CNV ≥ 0, *n* = 265), while the deletion group contained samples with low-CNV miR-3613 (CNV < 0, *n* = 242) (left). Kaplan–Meier survival curves of ER–negative breast cancer patients from TCGA database were depicted by their miR-3613 CNV (*P* = 0.52). (non-deletion group, *n* = 46; deletion group, *n* = 67) (right).

**Additional file 2: Supplementary Figure 2.** Histological analysis of breast cancer tissues with low or high miR-3613-3p expression by using hematoxylin-eosin staining.

**Additional file 3: Supplementary Figure 3.** Proliferation of MDA-MB-231 cells and apoptosis of MCF-7 cells. A) Proliferation of MDA-MB-231 cells transfected with miR-3613-3p or control mimic was analyzed by CCK-8 assay. B) Apoptosis of MCF-7 cells transfected with miR-3613-3p or control mimic was analyzed by flow cytometry.

**Additional file 4: Supplementary Figure 4.** The expression of stemness associated genes and miRNAs in tumor spheres of MDA-MB-231 cells. A) The expression of stemness genes (SOX2, OCT4, NANOG and LIN28B) were analyzed by RT-PCT in non-spheres or spheres of MDA-MB-231 cells. B) The expression of stemness associated miRNAs (let7 family and miR-146a) were analyzed by RT-PCT in non-spheres or spheres of MDA-MB-231 cells.

**Additional file 5: Supplementary Figure 5.** Frequency of cancer stem cells in MDA-MB-231 cells. A) Plot result of extreme limiting dilution analysis for MDA-MB-231 sphere formation. B) Frequency of CSCs was estimated based on MDA-MB-231 sphere formation assay.

**Additional file 6: Supplementary Figure 6.** Identification of miR-3613-3p target genes and related KEGG pathways. A) Venn diagram of predicted target genes miR-3613-3p from Targetscan and miRWalk online websites. B) Predicted 101 target genes of miR-3613-3p were enriched in KEGG pathways by DAVID online software.

**Additional file 7: Supplementary Figure 7.** Bioinformatic analysis of signaling pathways regulated by miR-3613-3p or its 3 target genes. A) Transcripts of differentially expressed genes were analyzed in two groups of breast cancer patients divided according to MIR3613 CNV from TCGA dataset (group of MIR3613 CNV ≥ 0, *n* = 586; group of MIR3613 CNV< 0, *n* = 492). Top 10 KEGG signaling pathways were shown according to above differentially expressed genes. B) Transcripts of differentially expressed genes were analyzed in two groups of breast cancer patients divided according to SMS, PAFAH1B2 and PDK3 expression from TCGA dataset (group of 3 target genes expression high, *n* = 166; group of 3 target genes expression low, *n* = 197). Top 10 KEGG signaling pathways were shown according to above differentially expressed genes.

**Additional file 8: Supplementary Figure 8.** The expression of proliferation related genes in breast cancer patients. Transcripts of differentially expressed genes were analyzed in two groups of breast cancer patients divided according to MIR3613 CNV from TCGA dataset (group of MIR3613 CNV ≥ 0, MIR3613 non-deletion, *n* = 586; group of MIR3613 CNV < 0, MIR3613 deletion, *n* = 492). The expression of CDC6, CDC25A, CDK1, ATM, E2F1 and MKI67 in above two groups were shown according to the transcriptome sequencing data.

**Additional file 9: Supplementary Figure 9.** Survival analysis of breast cancer patients based on the expression of SMS, PAFAH1B2 or PDK3. A) Kaplan-Meier survival curves of total, ER-negative or ER-positive breast cancer patients from TCGA database were depicted by SMS expression. B) Kaplan-Meier survival curves of total, ER-negative or ER-positive breast cancer patients from TCGA database were depicted by PAFAH1B2 expression. C) Kaplan-Meier survival curves of total, ER-negative or ER-positive breast cancer patients from TCGA database were depicted by PDK3 expression. D) Kaplan-Meier survival curves of total, ER-negative or ER-positive breast cancer patients from TCGA database were depicted by SMS, PAFAH1B2 and PDK3 expression.

**Additional file 10: Supplementary Figure 10.** Bioinformatic analysis of lncRNAs interacting with miR-3613-3p. A) Venn diagram of predicted targets from Targetscan and miRWalk online websites. B) The differential expression of 7 lncRNAs in breast cancer tissues (T, *n* = 1085) or normal tissues (N, *n* = 291) were analyzed by using the GEPIA database. C) Kaplan–Meier survival curves of breast cancer patients were depicted by the expression of NEAT1 (HR = 0.72 (0.52–0.98)) or SNHG16 (HR = 1.76 (1.28–2.42)), respectively. D) The expression of NEAT1 or SNHG16 in two separate group samples of breast cancer tissues from TCGA database divided by whether miR-3613 locus was deleted.

**Additional file 11: Supplementary Figure 11.** The description of binding sites of miR-3613-3p with its targets. A) The description of binding sites of miR-3613-3p with the 3′-UTR of SMS, PAFAH1B2 or PDK3. B) The description of binding sites of miR-3613-3p with the lncRNAs NEAT1 or SNHG16.

**Additional file 12: Supplementary Table 1.** Sequences of microRNAs. **Supplementary Table 2.** Primer sequences of mRNAs or microRNAs for RT-PCR. **Supplementary Table 3.** The correlation of miR-3613-3p expression clinicopathological characteristics of patients from breast cancer tissue array. **Supplementary Table 4.** Clinicopathological characteristics of patients from serologic detection.

## Data Availability

The TCGA and CCLE dataset are available in the UCSC Xena (https://xenabrowser.net). Bioinformatic analysis is based upon data generated by the DAVID Bioinformatics Tool (https://david.ncifcrf.gov), TargetScan (http://www.targetscan.org/vert_72), miRWalk (http://mirwalk.umm.uni-heidelberg.de), miRTarBase (http://mirtarbase.cuhk.edu.cn/php/index.php), DIANA-LncBase (http://carolina.imis.athena-innovation.gr/diana_tools/web/index.php?r=lncbasev2%2Findex), GEPIA (http://gepia.cancer-pku.cn), and Kaplan-Meier Plotter (http://kmplot.com/analysis). The data generated during the current study are available from the corresponding author on reasonable request.

## Abbreviations

CNVs: Copy number variations
TCGA: The Cancer Genome Atlas
CCLE: Broad Institute Cancer Cell Line Encyclopedia
qPCR: Quantitative polymerase chain reaction
lncRNA: Long non-coding RNA
UTR: Untranslated region
CSCs: Cancer Stem Cells
ATCC: American Type Culture Collection
GEPIA: Gene Expression Profiling Interactive Analysis
KEGG: Kyoto Encyclopedia of Genes and Genomes
H&E: Hematoxylin and eosin
