# Supplementary figures and images for: MicroRNA-3613-3p functions as a tumor suppressor and represents a novel therapeutic target in breast cancer
Source: Breast Cancer Res. 2021 Jan 25;23:12. doi: 10.1186/s13058-021-01389-9 (PMC7836180; doi:10.1186/s13058-021-01389-9)

A

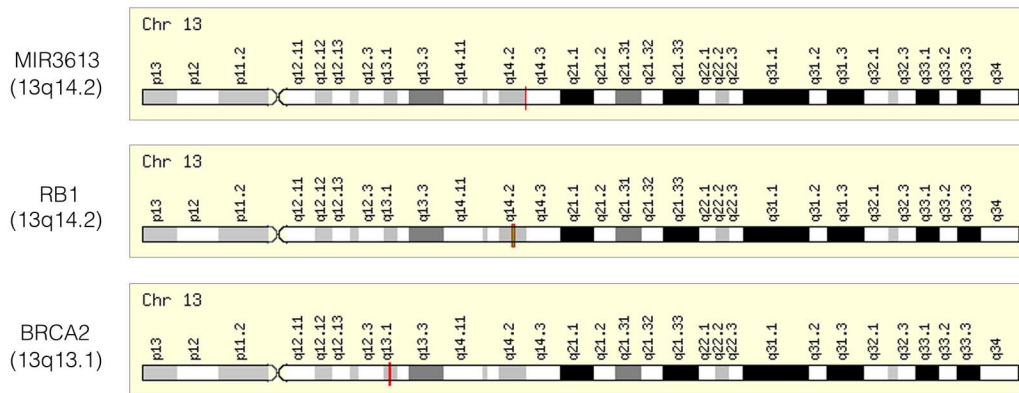

B

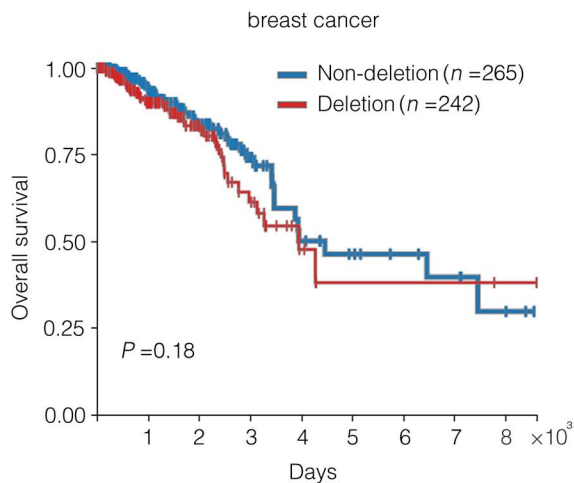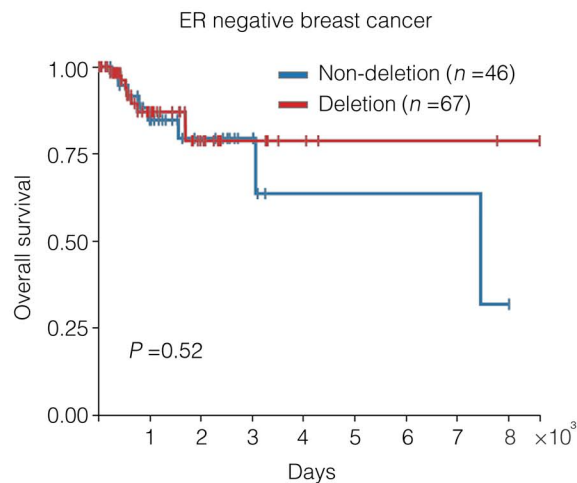

Supplement: Supplementary file 1 — Additional file 1: Supplementary Figure 1. The genomic location of MIR3613 and its CNV in breast cancer patients prognosis. A) The genomic location of MIR3613, RB1 and BRCA2 (red lines): bands according to Ensembl, locations according to GeneLoc. B) Kaplan–Meier survival curves of breast cancer patients from TCGA database were depicted by their genomic copy number value (CNV) of miR-3613 (P = 0.18). The non-deletion group contained samples with high-CNV miR-3613 (CNV ≥ 0, n = 265), while the deletion group contained samples with low-CNV miR-3613 (CNV < 0, n = 242) (left). Kaplan–Meier survival curves of ER–negative breast cancer patients from TCGA database were depicted by their miR-3613 CNV (P = 0.52). (non-deletion group, n = 46; deletion group, n = 67) (right). [file 13058_2021_1389_MOESM1_ESM.pdf]

high expression

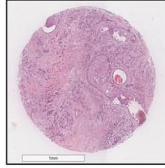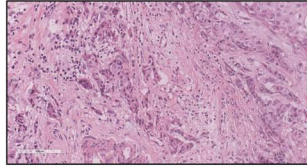

low expression

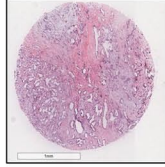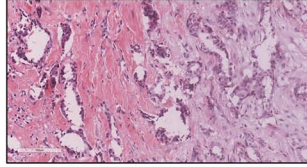

Supplement: Supplementary file 2 — Additional file 2: Supplementary Figure 2. Histological analysis of breast cancer tissues with low or high miR-3613-3p expression by using hematoxylin-eosin staining. [file 13058_2021_1389_MOESM2_ESM.pdf]

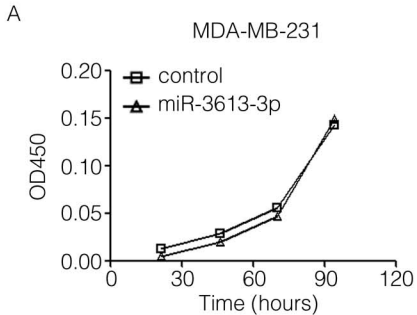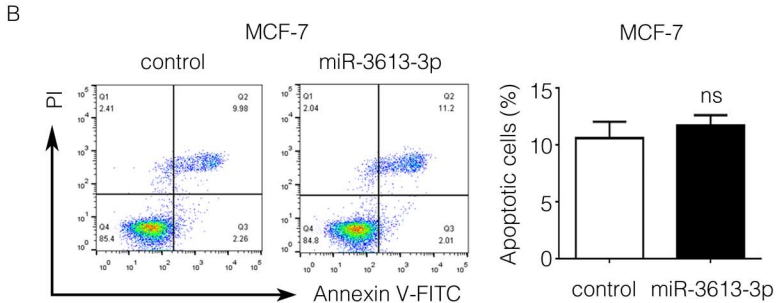

Supplement: Supplementary file 3 — Additional file 3: Supplementary Figure 3. Proliferation of MDA-MB-231 cells and apoptosis of MCF-7 cells. A) Proliferation of MDA-MB-231 cells transfected with miR-3613-3p or control mimic was analyzed by CCK-8 assay. B) Apoptosis of MCF-7 cells transfected with miR-3613-3p or control mimic was analyzed by flow cytometry. [file 13058_2021_1389_MOESM3_ESM.pdf]

A

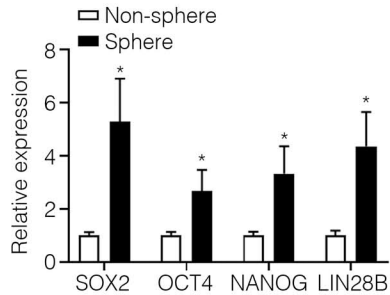

B

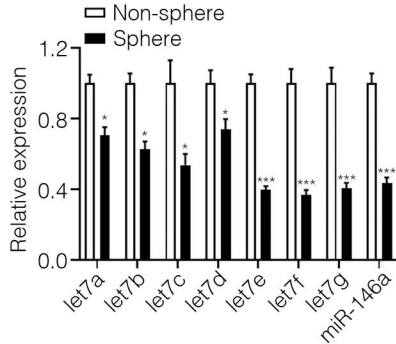

Supplement: Supplementary file 4 — Additional file 4: Supplementary Figure 4. The expression of stemness associated genes and miRNAs in tumor spheres of MDA-MB-231 cells. A) The expression of stemness genes (SOX2, OCT4, NANOG and LIN28B) were analyzed by RT-PCT in non-spheres or spheres of MDA-MB-231 cells. B) The expression of stemness associated miRNAs (let7 family and miR-146a) were analyzed by RT-PCT in non-spheres or spheres of MDA-MB-231 cells. [file 13058_2021_1389_MOESM4_ESM.pdf]

A

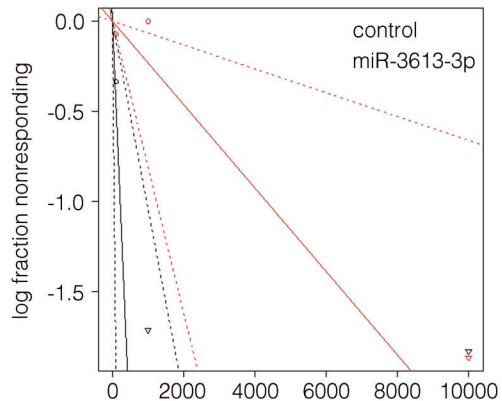

B

Confidence intervals for  $1 / (\text{stem cell frequency})$

| Group       | Lower | Estimate | Upper  |
|-------------|-------|----------|--------|
| control     | 962   | 217      | 49.2   |
| miR-3613-3p | 15139 | 4326     | 1236.7 |

Supplement: Supplementary file 5 — Additional file 5: Supplementary Figure 5. Frequency of cancer stem cells in MDA-MB-231 cells. A) Plot result of extreme limiting dilution analysis for MDA-MB-231 sphere formation. B) Frequency of CSCs was estimated based on MDA-MB-231 sphere formation assay. [file 13058_2021_1389_MOESM5_ESM.pdf]

A

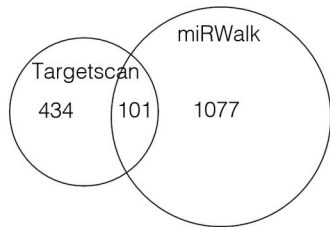

B

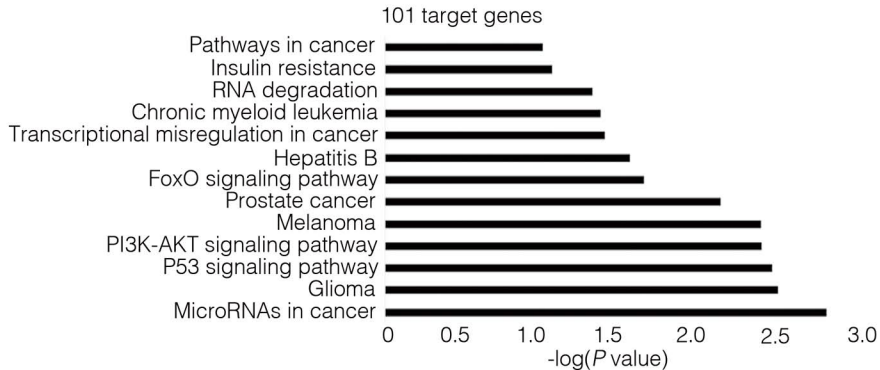

Supplement: Supplementary file 6 — Additional file 6: Supplementary Figure 6. Identification of miR-3613-3p target genes and related KEGG pathways. A) Venn diagram of predicted target genes miR-3613-3p from Targetscan and miRWalk online websites. B) Predicted 101 target genes of miR-3613-3p were enriched in KEGG pathways by DAVID online software. [file 13058_2021_1389_MOESM6_ESM.pdf]

A

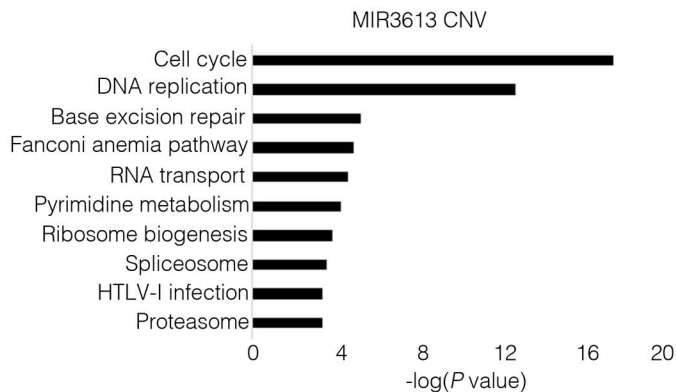

B

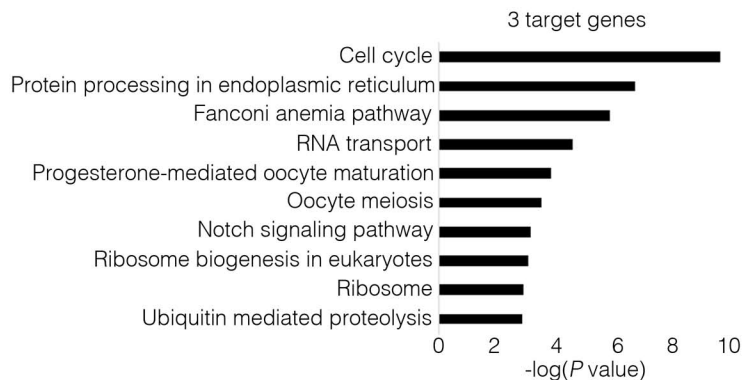

Supplement: Supplementary file 7 — Additional file 7: Supplementary Figure 7. Bioinformatic analysis of signaling pathways regulated by miR-3613-3p or its 3 target genes. A) Transcripts of differentially expressed genes were analyzed in two groups of breast cancer patients divided according to MIR3613 CNV from TCGA dataset (group of MIR3613 CNV ≥ 0, n = 586; group of MIR3613 CNV< 0, n = 492). Top 10 KEGG signaling pathways were shown according to above differentially expressed genes. B) Transcripts of differentially expressed genes were analyzed in two groups of breast cancer patients divided according to SMS, PAFAH1B2 and PDK3 expression from TCGA dataset (group of 3 target genes expression high, n = 166; group of 3 target genes expression low, n = 197). Top 10 KEGG signaling pathways were shown according to above differentially expressed genes. [file 13058_2021_1389_MOESM7_ESM.pdf]

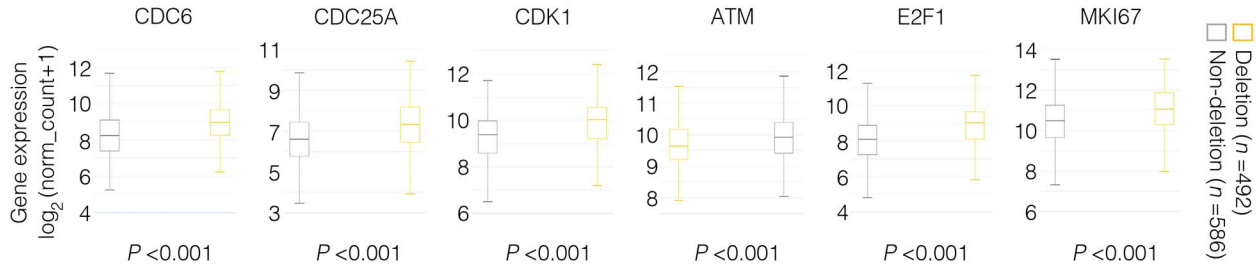

Supplement: Supplementary file 8 — Additional file 8: Supplementary Figure 8. The expression of proliferation related genes in breast cancer patients. Transcripts of differentially expressed genes were analyzed in two groups of breast cancer patients divided according to MIR3613 CNV from TCGA dataset (group of MIR3613 CNV ≥ 0, MIR3613 non-deletion, n = 586; group of MIR3613 CNV < 0, MIR3613 deletion, n = 492). The expression of CDC6, CDC25A, CDK1, ATM, E2F1 and MKI67 in above two groups were shown according to the transcriptome sequencing data. [file 13058_2021_1389_MOESM8_ESM.pdf]

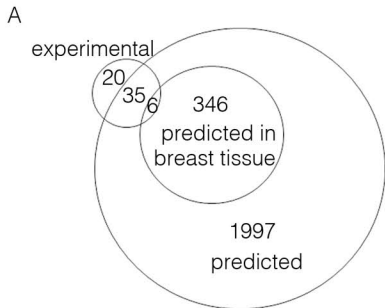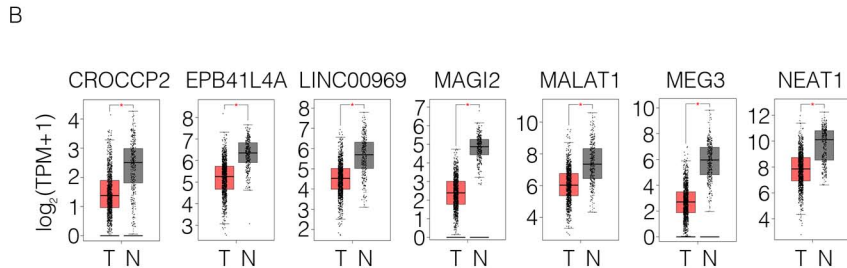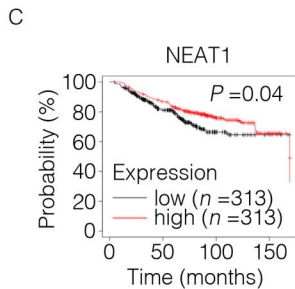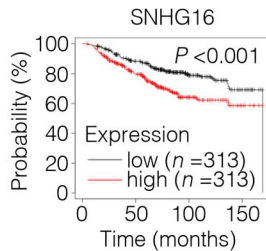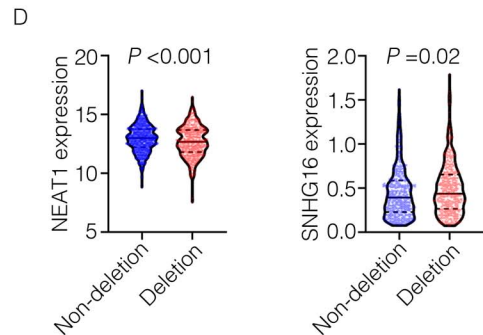

Supplement: Supplementary file 10 — Additional file 10: Supplementary Figure 10. Bioinformatic analysis of lncRNAs interacting with miR-3613-3p. A) Venn diagram of predicted targets from Targetscan and miRWalk online websites. B) The differential expression of 7 lncRNAs in breast cancer tissues (T, n = 1085) or normal tissues (N, n = 291) were analyzed by using the GEPIA database. C) Kaplan–Meier survival curves of breast cancer patients were depicted by the expression of NEAT1 (HR = 0.72 (0.52–0.98)) or SNHG16 (HR = 1.76 (1.28–2.42)), respectively. D) The expression of NEAT1 or SNHG16 in two separate group samples of breast cancer tissues from TCGA database divided by whether miR-3613 locus was deleted. [file 13058_2021_1389_MOESM10_ESM.pdf]
