# Supplementary material for: MicroRNA-3613-3p functions as a tumor suppressor and represents a novel therapeutic target in breast cancer
Source: Breast Cancer Res. 2021 Jan 25;23:12. doi: 10.1186/s13058-021-01389-9 (PMC7836180; doi:10.1186/s13058-021-01389-9)
Supplement: Supplementary file 9 — Additional file 9: Supplementary Figure 9. Survival analysis of breast cancer patients based on the expression of SMS, PAFAH1B2 or PDK3. A) Kaplan-Meier survival curves of total, ER-negative or ER-positive breast cancer patients from TCGA database were depicted by SMS expression. B) Kaplan-Meier survival curves of total, ER-negative or ER-positive breast cancer patients from TCGA database were depicted by PAFAH1B2 expression. C) Kaplan-Meier survival curves of total, ER-negative or ER-positive breast cancer patients from TCGA database were depicted by PDK3 expression. D) Kaplan-Meier survival curves of total, ER-negative or ER-positive breast cancer patients from TCGA database were depicted by SMS, PAFAH1B2 and PDK3 expression. [file 13058_2021_1389_MOESM9_ESM.pdf]

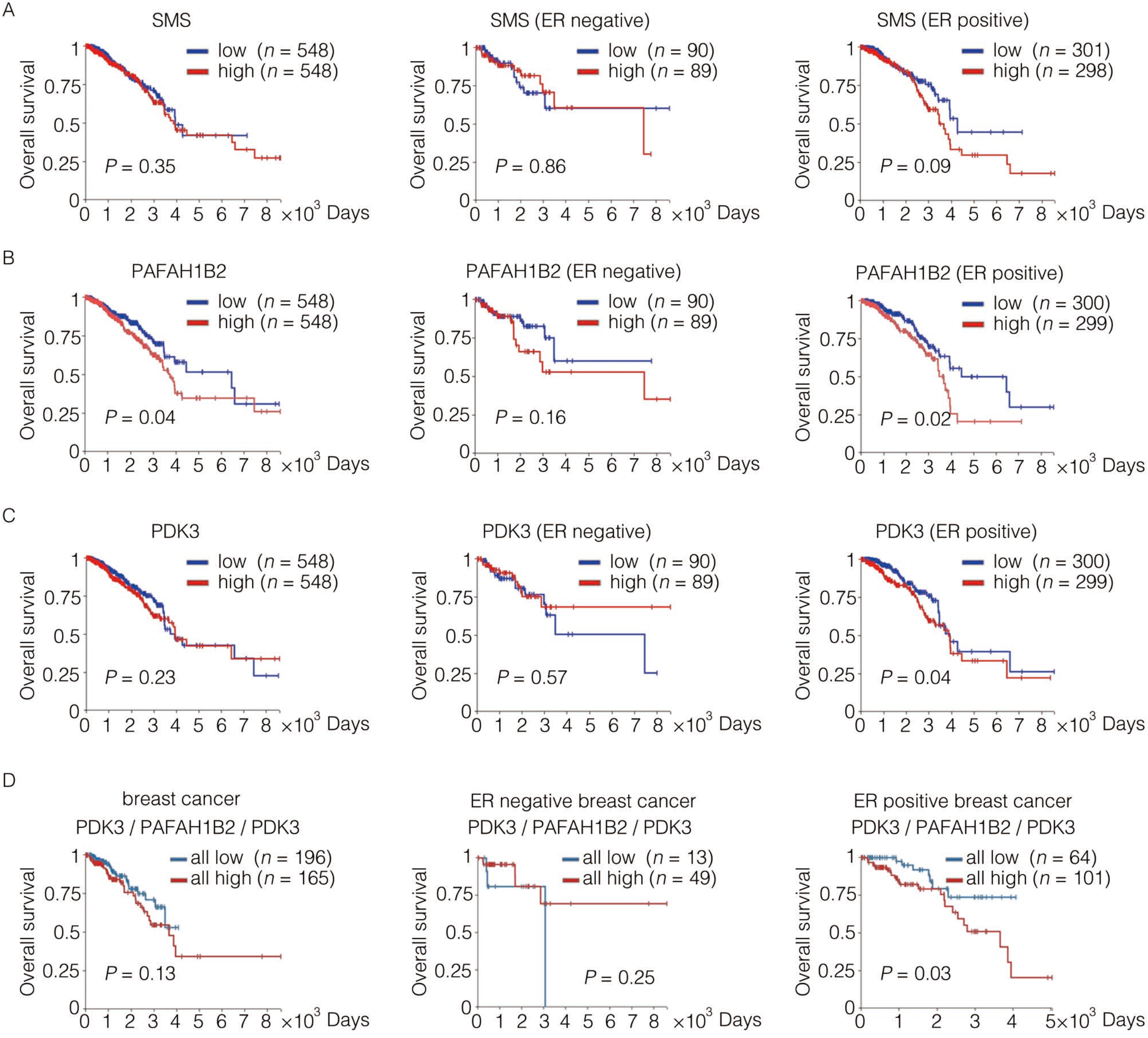
A
SMS
low (n = 548)
high (n = 548)
P = 0.35
SMS (ER negative)
low (n = 90)
high (n = 89)
P = 0.86
SMS (ER positive)
low (n = 301)
high (n = 298)
P = 0.09
B
PAFAH1B2
low (n = 548)
high (n = 548)
P = 0.04
PAFAH1B2 (ER negative)
low (n = 90)
high (n = 89)
P = 0.16
PAFAH1B2 (ER positive)
low (n = 300)
high (n = 299)
P = 0.02
C
PDK3
low (n = 548)
high (n = 548)
P = 0.23
PDK3 (ER negative)
low (n = 90)
high (n = 89)
P = 0.57
PDK3 (ER positive)
low (n = 300)
high (n = 299)
P = 0.04
D
breast cancer
PDK3 / PAFAH1B2 / PDK3
all low (n = 196)
all high (n = 165)
P = 0.13
ER negative breast cancer
PDK3 / PAFAH1B2 / PDK3
all low (n = 13)
all high (n = 49)
P = 0.25
ER positive breast cancer
PDK3 / PAFAH1B2 / PDK3
all low (n = 64)
all high (n = 101)
P = 0.03
Overall survival
×10^3 Days
