# Supplementary material for: MicroRNA-3613-3p functions as a tumor suppressor and represents a novel therapeutic target in breast cancer
Source: Breast Cancer Res. 2021 Jan 25;23:12. doi: 10.1186/s13058-021-01389-9 (PMC7836180; doi:10.1186/s13058-021-01389-9)
Supplement: Supplementary file 11 — Additional file 11: Supplementary Figure 11. The description of binding sites of miR-3613-3p with its targets. A) The description of binding sites of miR-3613-3p with the 3′-UTR of SMS, PAFAH1B2 or PDK3. B) The description of binding sites of miR-3613-3p with the lncRNAs NEAT1 or SNHG16. [file 13058_2021_1389_MOESM11_ESM.pdf]

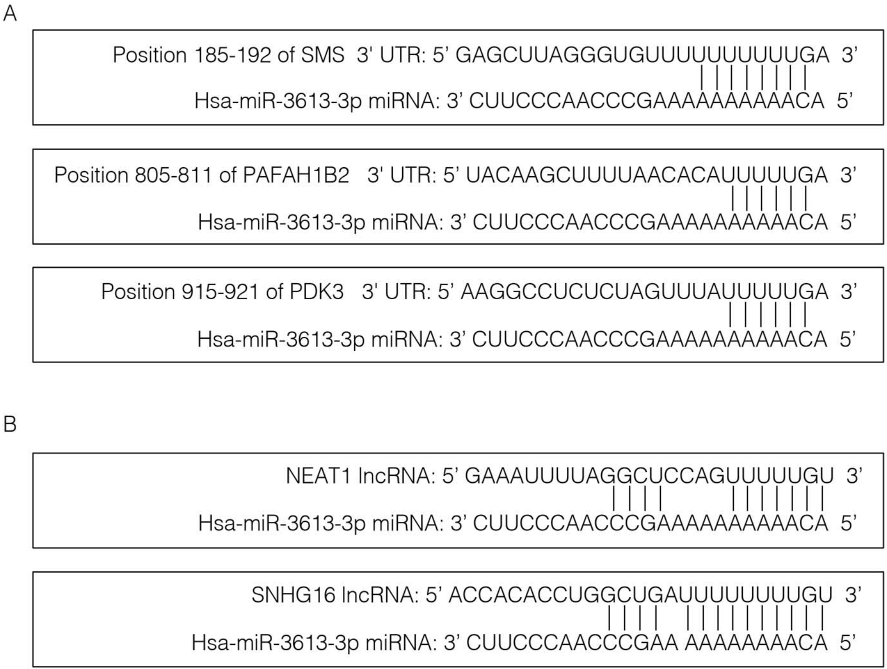
A
Position 185-192 of SMS 3' UTR: 5’ GAGCUUAGGGUGUUUUUUUUUGA 3’
Hsa-miR-3613-3p miRNA: 3’ CUUCCCAACCCGAAAAAAAAAACA 5’
Position 805-811 of PAFAH1B2 3' UTR: 5’ UACAAGCUUUUAACACAUUUUUGA 3’
Hsa-miR-3613-3p miRNA: 3’ CUUCCCAACCCGAAAAAAAAAACA 5’
Position 915-921 of PDK3 3' UTR: 5’ AAGGCCUCUCUAGUUUAUUUUUGA 3’
Hsa-miR-3613-3p miRNA: 3’ CUUCCCAACCCGAAAAAAAAAACA 5’
B
NEAT1 lncRNA: 5’ GAAAUUUUAGGCUCCAGUUUUUGU 3’
Hsa-miR-3613-3p miRNA: 3’ CUUCCCAACCCGAAAAAAAAAACA 5’
SNHG16 lncRNA: 5’ ACCACACCUGGCUGAUUUUUUUUGU 3’
Hsa-miR-3613-3p miRNA: 3’ CUUCCCAACCCGAA AAAAAAAACA 5’
