# Supplementary material for: MicroRNA-3613-3p functions as a tumor suppressor and represents a novel therapeutic target in breast cancer
Source: Breast Cancer Res. 2021 Jan 25;23:12. doi: 10.1186/s13058-021-01389-9 (PMC7836180; doi:10.1186/s13058-021-01389-9)
Supplement: Supplementary file 12 — Additional file 12: Supplementary Table 1. Sequences of microRNAs. Supplementary Table 2. Primer sequences of mRNAs or microRNAs for RT-PCR. Supplementary Table 3. The correlation of miR-3613-3p expression clinicopathological characteristics of patients from breast cancer tissue array. Supplementary Table 4. Clinicopathological characteristics of patients from serologic detection. [file 13058_2021_1389_MOESM12_ESM.doc]

Supplementary Table 1

Sequences of microRNAs

| microRNA | Sequence (5’-3’) |
| --- | --- |
| hsa-miR-3613-3p | ACAAAAAAAAAAGCCCAACCCUUC |
| hsa-let-7a-5p | UGAGGUAGUAGGUUGUAUAGUU |
| hsa-let-7b-5p | UGAGGUAGUAGGUUGUGUGGUU |
| hsa-let-7c-5p | UGAGGUAGUAGGUUGUAUGGUU |
| hsa-let-7d-5p | AGAGGUAGUAGGUUGCAUAGUU |
| hsa-let-7e-5p | UGAGGUAGGAGGUUGUAUAGUU |
| hsa-let-7f-5p | UGAGGUAGUAGAUUGUAUAGUU |
| hsa-let-7g-5p | UGAGGUAGUAGUUUGUACAGUU |
| hsa-miR-146a-5p | UGAGAACUGAAUUCCAUGGGUU |
| cel-miR-39-3p | UCACCGGGUGUAAAUCAGCUUG |

Supplementary Table 2

Primer sequences of mRNAs or microRNAs for RT-PCR

| Primer | Sequence (5’-3’) |
| --- | --- |
| SOX2 Forward | AAAACAGCCCGGACCGCGTC |
| SOX2 Reverse | CTCGTCGATGAACGGCCGCT |
| OCT4 Forward | AAGCGATCAAGCAGCC |
| OCT4 Reverse | GGAAAGGGACCGAGGAGTA |
| NANOG Forward | ACCTCAGCCTCCAGCAGATGCA |
| NANOG Reverse | GGTGCTGAGGCCTTCTGCGT |
| LIN28B Forward | CATCTCCATGATAAACCGAGAGG |
| LIN28B Reverse | GTTACCCGTATTGACTCAAGGC |
| ACTIN Forward | TCATGAAGTGTGACGTGGACATC |
| ACTIN Reverse | CAGGAGGAGCAATGATCTTGATCT |
| hsa-miR-3613-3p RT | GTCGTATCCAGTGCAGGGTCCGAGGTATTCGCACTGGATACGACGAAGGG |
| hsa-miR-3613-3p Forward | GGACAAAAAAAAAAGCCCAA |
| hsa-let-7a-5p RT | GTCGTATCCAGTGCAGGGTCCGAGGTATTCGCACTGGATACGACAACTAT |
| hsa-let-7a-5p Forward | GCGTGAGGTAGTAGGTTGT |
| hsa-let-7b-5p RT | GTCGTATCCAGTGCAGGGTCCGAGGTATTCGCACTGGATACGACAACCAC |
| hsa-let-7b-5p Forward | GCGTGAGGTAGTAGGTTGT |
| hsa-let-7c-5p RT | GTCGTATCCAGTGCAGGGTCCGAGGTATTCGCACTGGATACGACAACCAT |
| hsa-let-7c-5p Forward | GCGTGAGGTAGTAGGTTGT |
| hsa-let-7d-5p RT | GTCGTATCCAGTGCAGGGTCCGAGGTATTCGCACTGGATACGACAACTAT |
| hsa-let-7d-5p Forward | GCGAGAGGTAGTAGGTTGC |
| hsa-let-7e-5p RT | GTCGTATCCAGTGCAGGGTCCGAGGTATTCGCACTGGATACGACAACTAT |
| hsa-let-7e-5p Forward | GCGTGAGGTAGGAGGTTG |
| hsa-let-7f-5p RT | GTCGTATCCAGTGCAGGGTCCGAGGTATTCGCACTGGATACGACAACTAT |
| hsa-let-7f-5p Forward | GCGCTGAGGTAGTAGATTGT |
| hsa-let-7g-5p RT | GTCGTATCCAGTGCAGGGTCCGAGGTATTCGCACTGGATACGACAACTGT |
| hsa-let-7g-5p Forward | GCGCTGAGGTAGTAGTTTGT |
| hsa-miR-146a-5p RT | GTCGTATCCAGTGCAGGGTCCGAGGTATTCGCACTGGATACGACAACCCA |
| hsa-miR-146a-5p Forward | GCGCTGAGAACTGAATTCCA |
| Universal Reverse | CAGTGCAGGGTCCGAGGT |
| U6 RT | AAAATATGGAACGCTTCACGAATTTG |
| U6 Forward | CTCGCTTCGGCAGCACATATACT |
| U6 Reverse | ACGCTTCACGAATTTGCGTGTC |
| cel-miR-39 RT | GTCGTATCCAGTGCAGGGTCCGAGGTATTCGCACTGGATACGACCAAGCT |

RT: Reverse transcription

Supplementary Table 3

The correlation of miR-3613-3p expression clinicopathological characteristics of patients from breast cancer tissue array

| Characteristics | Patients (*n* = 30) | |
| --- | --- | --- |
| Histologic type |  | |
| Invasive ductal carcinoma | *n* = 30 | |
| miR-3613-3p expression | Low (*n*) | High (*n*) |
| Pathology staging |  |  |
| Ⅰ | 3 | 1 |
| Ⅱa | 9 | 3 |
| Ⅱb | 5 | 2 |
| Ⅲa | 3 | 0 |
| Ⅲc | 1 | 3 |
| Lymph node metastasis |  |  |
| N0 | 12 | 4 |
| N1 | 6 | 2 |
| N2 | 2 | 0 |
| N3 | 1 | 3 |

Supplementary Table 4

Clinicopathological characteristics of patients from serologic detection

| Characteristics | Patients (*n* = 20) |
| --- | --- |
| Histologic type |  |
| Invasive ductal carcinoma | *n* = 20 |
| Clinical staging |  |
| 1 | *n* = 5 |
| 2 | *n* = 7 |
| 3 | *n* = 8 |
| Tumor size |  |
| T1 | *n* = 6 |
| T2 | *n* = 11 |
| T3 | *n* = 2 |
| Tx | *n* = 1 |
| Lymph node metastasis |  |
| N0 | *n* = 13 |
| N1 | *n* = 1 |
| N2 | *n* = 4 |
| N3 | *n* = 2 |
